# Inhibition of the STAT3 signaling pathway contributes to apigenin-mediated anti-metastatic effect in melanoma

**DOI:** 10.1038/srep21731

**Published:** 2016-02-25

**Authors:** Hui-Hui Cao, Jian-Hong Chu, Hiu-Yee Kwan, Tao Su, Hua Yu, Chi-Yan Cheng, Xiu-Qiong Fu, Hui Guo, Ting Li, Anfernee Kai-Wing Tse, Gui-Xin Chou, Huan-Biao Mo, Zhi-Ling Yu

**Affiliations:** 1 Consun Chinese Medicines Research Centre for Renal Diseases, School of Chinese Medicine, Hong Kong Baptist University, Hong Kong, China; 2 School of Traditional Chinese Medicine, Southern Medical University, Guangzhou, P.R. China; 3 The MOE Key Laboratory for Standardization of Chinese Medicines, Institute of Chinese Materia Medica, Shanghai University of Traditional Chinese Medicine, Shanghai, P.R. China; 4 Department of Nutrition, Georgia State University, Georgia, USA

## Abstract

Signal transducer and activator of transcription 3 (STAT3) signaling is constantly activated in human melanoma, and promotes melanoma metastasis. The dietary flavonoid apigenin is a bioactive compound that possesses low toxicity and exerts anti-metastatic activity in melanoma. However, the anti-metastasis mechanism of apigenin has not been fully elucidated. In the present study, we showed that apigenin suppressed murine melanoma B16F10 cell lung metastasis in mice, and inhibited cell migration and invasion in human and murine melanoma cells. Further study indicated that apigenin effectively suppressed STAT3 phosphorylation, decreased STAT3 nuclear localization and inhibited STAT3 transcriptional activity. Apigenin also down-regulated STAT3 target genes MMP-2, MMP-9, VEGF and Twist1, which are involved in cell migration and invasion. More importantly, overexpression of STAT3 or Twist1 partially reversed apigenin-impaired cell migration and invasion. Our data not only reveal a novel anti-metastasis mechanism of apigenin but also support the notion that STAT3 is an attractive and promising target for melanoma treatment.

Melanoma is the most serious type of skin cancer because it is highly aggressive and can spread earlier and more quickly than other skin cancers. It represents only 4% of the skin cancer cases, but it accounts for more than 80% of all skin cancer-related deaths^1^. Surgical resection can only cure early diagnosed primary melanoma^2^. For the advanced melanoma, current therapeutics such as BRAF inhibitors, cytotoxic T lymphocyte-associated antigen 4 antibody and interleukin-2 biological therapy show different toxicities and side-effects^3^,^4^,^5^. Resistance to the BRAF targeted therapy in melanoma has also been reported, and has become a major problem for BRAF inhibitors^6^. Therefore, novel targeted therapies with low toxicity are urgently needed.

Transcription factor STAT3 (signal transducer and activator of transcription 3) is constitutively activated in 50–90% of melanomas^7^. Activation of STAT3 promotes transcription of many genes that involve in melanoma metastasis^7^. Examinations show that activation of STAT3 in human melanoma promotes brain metastasis, and increases the expressions of vascular endothelial growth factor (VEGF) and matrix metalloproteinase-2 (MMP-2)^8^. Constitutive STAT3 activation has been reported to up-regulate VEGF expression and stimulate melanoma tumor angiogenesis^9^. In addition, a wide array of aberrated oncogenes/angiogenic proteins such as Src^10^ and JAK2^11^ converges on STAT3. Therefore, targeting STAT3 assaults melanoma on multiple fronts by suppressing the oncogenic potentials due to upstream and downstream aberrations^12^. Indeed, studies show that inhibiting STAT3 signaling in melanoma tumor models prevents metastasis^8^,^13^ and inhibits angiogenesis^14^.

Accumulating evidences from observational and prospective studies indicate that natural compounds present in the human diet or as supplements may substantially alter the natural history of carcinogenesis^15^. In recent years, there has been growing interest in the role of nutrition in melanoma chemoprevention^16^. Apigenin is a natural dietary flavonoid that has multiple biological functions such as anti-inflammatory and anti-oxidant properties^17^. Moreover, mounting epidemiological evidence suggests that intake of apigenin and other flavonoids reduces risk of cancers and this dietary intervention is negatively associated with the cancer recurrence^17^. Early in 1997, it has been found that apigenin inhibited ultraviolet light-induced skin carcinogenesis in mice^18^. Subsequent studies also suggest the anti-melanoma effects of apigenin, which include inhibition of melanoma metastasis^19^,^20^. Some of the cellular targets for apigenin have been identified^21^, including STAT3 signaling^22^,^23^. However, whether apigenin affects STAT3 signaling in melanoma metastasis has not been elucidated. In the present study, we examined the involvement of the STAT3 signaling pathway in the anti-metastatic effect of apigenin in melanoma. Two human melanoma cell lines A375 and G361 with constitutive activation of STAT3^24^, together with a murine melanoma cell line B16F10 that widely used in lung metastasis study^25^ were employed in this study.

## Results

### Apigenin inhibited melanoma B16F10 cell lung metastasis

**Figure 1:**
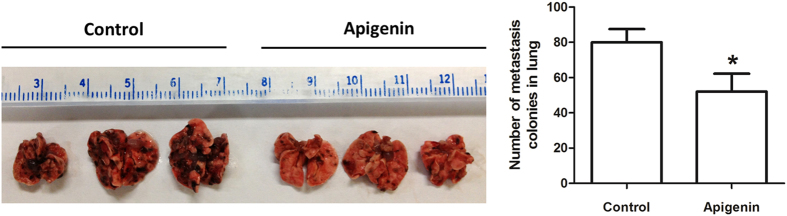
Apigenin inhibited murine melanoma B16F10 cell lung metastasis. B16F10 melanoma cells were injected into the tail vein of the C57BL/6 mice. These mice then received intragastric administration of vehicle or apigenin (150 mg/kg/day) for 24 consecutive days. Lung metastasis of B16F10 melanoma cells in the mouse model (upper) and the metastasis nodules number in the lungs (bottom) were shown. Data were mean ± SD, *n* = 8, *P < 0.05.

We first determined the *in vivo* anti-metastatic effect of apigenin in an experimental lung metastasis model. B16F10 melanoma cells were injected into the lateral tail vein of C57BL/6 mice. Then these mice were treated by intragastric administration with either vehicle (0.5% CMC-Na solution) or apigenin (150 mg/kg)^26^. We found that the apigenin-treated mice had significant fewer metastatic nodules (Fig. 1) when compared to the vehicle control group, suggesting apigenin inhibits the metastasis potential of B16F10 melanoma cells *in vivo* in our mouse model. This finding is in agreement with a previous report^20^.

### Apigenin impaired the migratory and invasive abilities of melanoma cells

**Figure 2:**
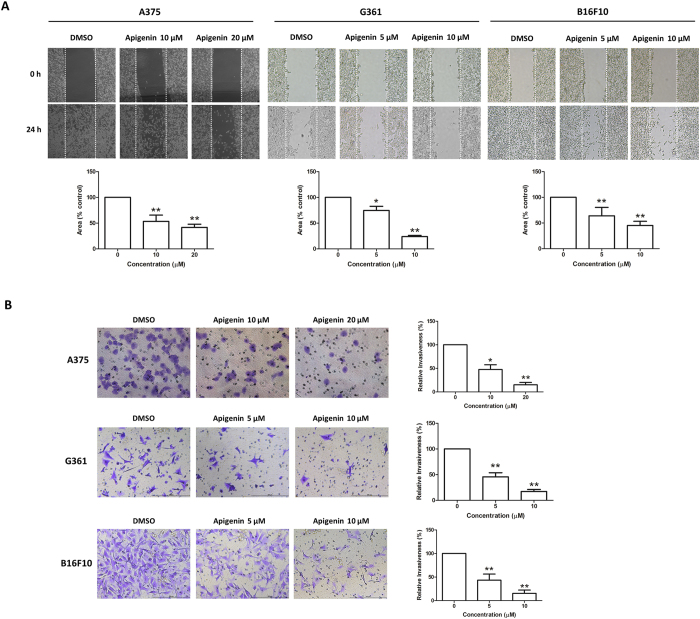
Apigenin inhibited melanoma cell migration and invasion. (**A**) A single scratch was created in the confluent monolayer of A375, G361 or B16F10 cells. The scratch was photographed at 0 h and 24 h after treatment with the indicated concentrations of apigenin (upper) and relative migrated areas (bottom) were analyzed by Image J software. (**B**) A375, G361 or B16F10 cells were treated with indicated concentrations of apigenin for 16 h, and the invasive capacity was determined by the Transwell invasion assay. Representative photographs of invasive cells (left) and quantification of invasive cells (right) were shown. Data were mean ± SD from three independent experiments, *P < 0.05 and **P < 0.01.

Apigenin has been shown to inhibit tumor cell migration and invasion in various types of cancers^27^,^28^, including mouse melanoma B16-BL6 cells^19^; however, its effects in human melanoma cells remain unknown. We therefore examined the effects of apigenin on migratory and invasive capacities of melanoma cells by using the wound healing assay and Transwell invasion assay, respectively. As shown in Fig. 2, apigenin (10 and 20 μM) significantly inhibited A375 cell motility and invasion in dose-dependent manners. It was reported that the migratory ability of A375 was about two times higher than that of G361 cells^29^. In agreement with this, we also found that the migratory and invasive capacities of G361 were much lower than A375 cells. Nevertheless, apigenin (5 and 10 μM) showed significant inhibitory effects on G361 cell migration and invasion. Apigenin (5 and 10 μM) also dose-dependently inhibited B16F10 cell migration and invasion. These data suggest that apigenin inhibits melanoma cells migration and invasion. Under the same culture condition, apigenin did not apparently affect cell viability (see Supplementary Fig. 1).

### Apigenin reduced constitutive STAT3 phosphorylation, suppressed STAT3 nuclear localization and STAT3-luciferase reporter activity in melanoma cells

**Figure 3:**
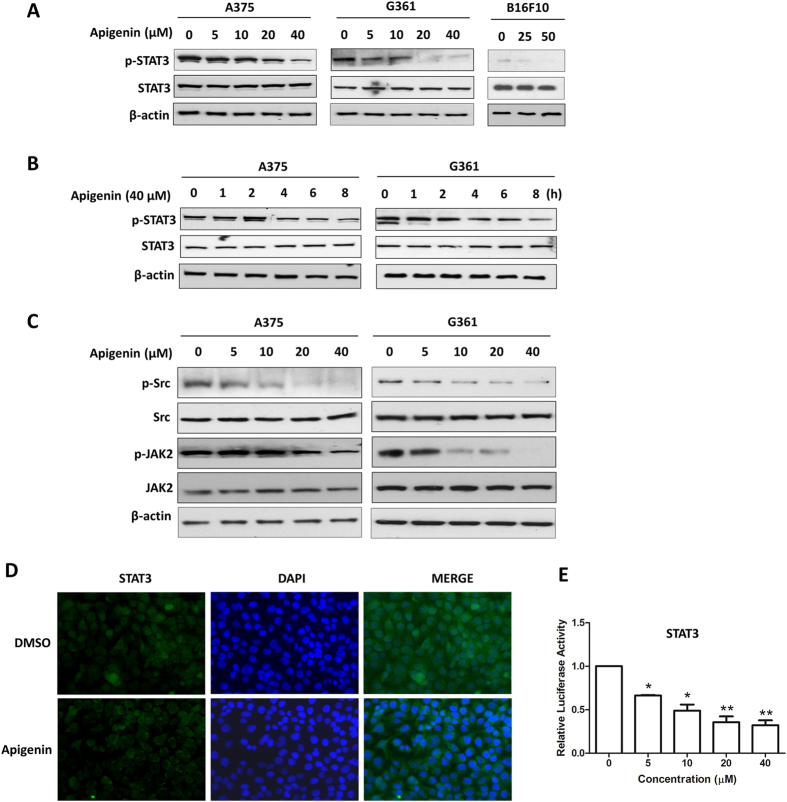
Apigenin reduced constitutive STAT3 phosphorylation, suppressed STAT3 nuclear localization and STAT3-luciferase reporter activity in melanoma cells. Cells were treated with apigenin (**A**) at different concentrations for 24 h or (**B**) at 40 μM for different time periods as indicated. Then the expression levels of STAT3 and phospho-STAT3 were determined by the Western blot analysis. (**C**) Cells were treated with apigenin at various concentrations for 8 h and then the expressions of phospho-JAK2, JAK2, phospho-Src and Src were examined by Western blotting. (**D**) A375 cells were treated with 20 μM apigenin for 8 h, and then analyzed for the intracellular distribution of STAT3 by immunocytochemistry. (**E**) A375 cells were transfected with a STAT3-luciferase reporter plasmid together with a PRL-CMV construct for 12 h, followed by treatment with indicated concentrations of apigenin for another 12 h, and then the STAT3 transcriptional activity was measured by the dual-luciferase reporter assay. Data were shown as mean ± SD from three independent experiments, *P < 0.05 and **P < 0.01.

Constitutive activation of the STAT3 signaling pathway is well recognized to play a critical role in human melanoma development and progression by promoting cancer cell growth, survival, migration and invasion^7^. Therefore, we examined if apigenin inhibited the constitutive phosphorylation/activation of STAT3. As shown in Fig. 3A, in melanoma A375, G361 and B16F10 cells, the phosphorylation of STAT3 at the tyrosine 705 (Tyr705) site was dose-dependently inhibited by apigenin (Fig. 3A). When treated melanoma cells with 40 μM apigenin for various durations, the expression levels of phospho-STAT3 were decreased at time dependent manners (Fig. 3B); while apigenin did not affect the total STAT3 protein expressions (Fig. 3A,B) at the same concentration. These findings strongly suggest that apigenin effectively suppresses the constitutive activation of STAT3 in melanoma cells.

STAT3 has been reported to be activated by tyrosine kinases of the Janus family (JAKs) and Src kinase families at tyrosine 705 (Tyr705) site^30^. So we wondered if apigenin inhibited the activation of JAK2 and Src. As shown in Fig. 3C, apigenin dose-dependently suppressed the constitutive phosphorylation of JAK2 and Src in these two cell types. The expression levels of total JAK2 and Src kinase were not affected by apigenin treatment under the same condition. These results suggest that inhibition of STAT3 activation by apigenin is, at least in part, attributed to the reduction of the phosphorylation of JAK2 and Src in melanoma cells.

Tyrosine phosphorylation of STAT3 at Tyr705 initiates STAT3 dimerization and nuclear translocation. So we examined whether apigenin inhibited the nuclear translocation of STAT3. As shown in Fig. 3D, apigenin treatment obviously reduced STAT3 nuclear localization. STAT3 is a transcription factor. Inhibition of its nuclear localization will impede its binding on gene promoters and the subsequent gene transcriptional activities. To examine whether apigenin affected STAT3-mediated transcriptional activity, a STAT3-luciferase reporter construct (4xM67 pTATA TK-Luc) harboring four copies of STAT3 binding sites was transfected into A375 cells. Then these cells were treated with indicated concentrations of apigenin for 12 h before measuring the transcriptional activity by luciferase assay. As shown in Fig. 3E, apigenin significantly inhibited the STAT3-luciferase reporter activity in a dose-dependent manner. These results further confirm the inhibitory effect of apigenin on STAT3 signaling.

### Apigenin regulated the expressions of STAT3 target genes involved in cell migration and invasion

**Figure 4:**
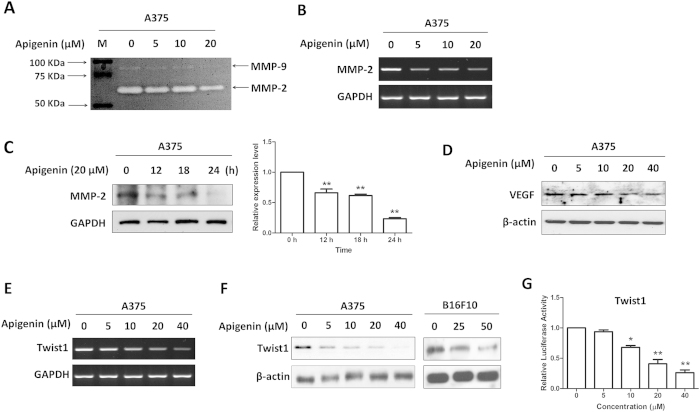
Apigenin regulated the expression of STAT3 target genes. Cells were treated with various concentrations of apigenin for 24 h, and then (**A**) the enzymatic activities of MMP-2 and MMP-9 were determined by gelatinase zymography, (**B**) the mRNA expression level of MMP-2 was measured by RT-PCR, the protein levels of (**C**) MMP-2 and (**D**) VEGF were determined by immunoblotting, the relative protein level of MMP-2 was analyzed by Image J software and shown as mean ± S.D., **P < 0.01. (**E**) Twsit1 mRNA expression level and (**F**) Twist1 protein expression level were determined by RT-PCR and Western blotting, respectively. (**G**) A375 cells were transfected with a Twist1 promoter construct together with a PRL-CMV construct for 12 h, and then exposed to various concentrations of apigenin for another 12 h. The Twist1 transcriptional activity was measured by the dual-luciferase reporter assay. Data were shown as mean ± SD from three independent experiments, *P < 0.05 and **P < 0.01.

We next examined if apigenin affected STAT3 target genes in human melanoma cells. MMP-2 and MMP-9 are the target genes of STAT3 involving in cell migration and invasion^8^,^13^. As shown in Fig. 4A, apigenin inhibited the gelatinase activities of MMP-2 and MMP-9 in A375 cells. Apigenin also dose-dependently reduced the mRNA expression level of MMP-2 (Fig. 4B) and decreased its protein level in a time dependent manner (Fig. 4C), while the mRNA level of MMP-9 was too low to be detected. VEGF, another STAT3 target gene^9^, is one of the most potent mediators of tumor angiogenesis and metastasis. Apigenin also dose-dependently reduced the protein level of VEGF (Fig. 4D).

Twist1 is a transcription factor that frequently expressed in a wide array of human cancers including melanoma. It correlates with aggressive, invasive and metastatic lesions^31^. It was reported that constitutive activation of STAT3 can significantly activate the Twist1 promoter^32^. We found that apigenin reduced the Twist1 mRNA (Fig. 4E) and protein (Fig. 4F) levels in dose-dependent manners. To ascertain whether the decreased Twist1 mRNA expression was due to an inhibition of the Twist1 promoter activity, we generated a Twist1-promoter reporter construct (Twist1-Luc) and performed the luciferase assay. Data showed that apigenin reduced Twist1 promoter activity in a dose-dependent manner (Fig. 4G).

### Apigenin treatment partially reversed epithelial-to-mesenchymal transition (EMT)

**Figure 5:**
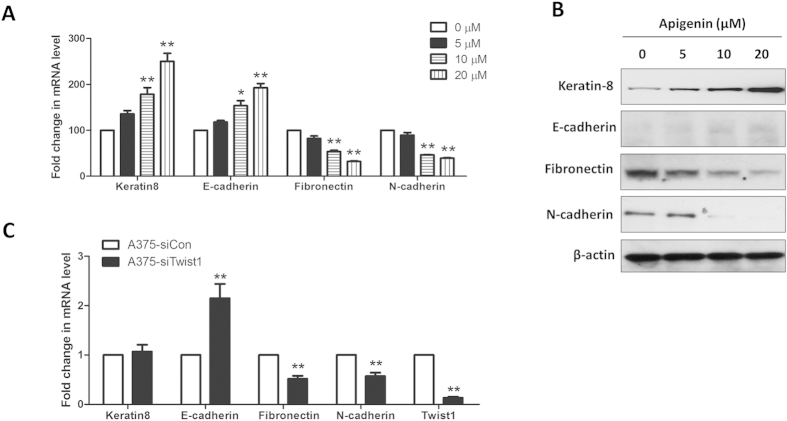
Apigenin treatment partially reversed EMT in A375 cells. (**A**) A375 cells were treated with indicated concentrations of apigenin for 24 h, and the mRNA expression levels of several EMT markers were determined by real-time PCR. Data were shown as mean ± SD from three independent experiments, *P < 0.05 and **P < 0.01. (**B**) After apigenin treatment for 24 h, the protein expression levels of EMT markers in A375 cells were determined by Western blotting. (**C**) A375 cells were transfected with a Twist1-specific siRNA (si-Twist1) or a control siRNA (si-Con) for 24 h, the mRNA expression levels of several EMT markers were determined by real-time PCR. Data were shown as mean ± SD from three independent experiments, **P < 0.01.

Twist1 has also been reported to induce epithelial-to-mesenchymal transition (EMT)^33^. EMT is a biological process that allows epithelial cells to undergo multiple biochemical changes that enable them to assume a mesenchymal cell phenotype. This process usually includes enhanced migratory and invasive capacities^34^. Tumors derived from epithelial cells can become more motile and invasive by acquiring characteristics of mesenchymal cells^35^. Since apigenin inhibited the expression level and promoter activity of Twist1 in melanoma A375 cells, we wondered if apigenin reversed the EMT in A375 cells. Real-time PCR analysis demonstrated that apigenin treatment dose-dependently increased the mRNA expression levels of epithelial markers keratin 8 and E-cadherin, while decreased the levels of mesenchymal markers fibronectin and N-cadherin (Fig. 5A). Western blot analysis also showed that apigenin caused partial reversal of EMT in melanoma cells, as evidenced by upregulation of keratin 8 and E-cadherin, and downregulation of fibronectin and N-cadherin (Fig. 5B). The partial reversal of EMT in melanoma cells after apigenin treatment may due to a reduction in Twist1 expression. Because in the Twist1-siRNA transfected cells (A375-siTwist1), mRNA levels of fibronectin and N-cadherin were reduced and that of an epithelial marker E-cadherin was increased (Fig. 5C). These data suggest that apigenin treatment or reduced expression of Twist1 regulates the expressions of the EMT factors and hence partially reverses the EMT in melanoma cells.

### Overexpression of Twist1 abrogated apigenin-mediated inhibitory effects on melanoma cell migration and invasion

**Figure 6:**
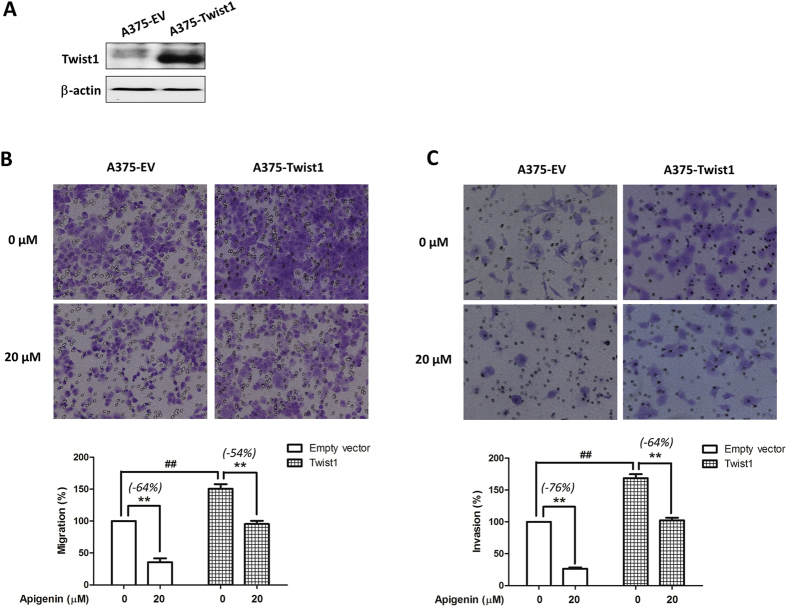
Overexpression of Twist1 abrogated apigenin-mediated migration and invasion inhibitions in A375 cells. A375 cells were transiently transfected with an empty vector or a Twist1-expressing construct for 24 h, and then (**A**) the expression level of Twist1 in A375-Twist1 cells and A375-EV cells were examined by immunoblotting. (**B,C**) A375-EV or A375-Twist1 cells were incubated with 20 μM apigenin for 16 h, and (**B**) cell migratory and (**C**) invasive abilities were measured by the Transwell chamber assay. Representative photographs of migrated or invasive cells (upper) and quantification of these cells (bottom) were shown. Data were mean ± SD from three independent experiments, ^##^*P* < 0.01 and **P < 0.01.

To further investigate if Twist1 involved in apigenin-mediated migration and invasion inhibition in melanoma cells, we overexpressed Twist1 in A375 cells by transient transfection of a Twist1-expressing construct pcDNA3.1-Twist1. After 24 h transfection, the expression of Twist1 was increased remarkably (Fig. 6A). Moreover, cells that transfected with Twist1 construct showed a significant increase in the migratory (Fig. 6B) and invasive (Fig. 6C) abilities as compared with cells that were transfected with the empty vector (^##^*P* < 0.01). In Twist1 overexpressing cells, apigenin-mediated cell migration inhibition was partially reduced from 64% to 54% (Fig. 6E), and cell invasion inhibition was reduced from 76% to 64% (Fig. 6F). All these data suggest that the inhibitory effects of apigenin on melanoma migration and invasion are due to, at least in part, the reduction of Twist1 expression.

### Ectopic expression of STAT3 rescued apigenin-mediated melanoma cell migration and invasion, and reduced the apigenin-mediated Twist 1 inhibition

**Figure 7:**
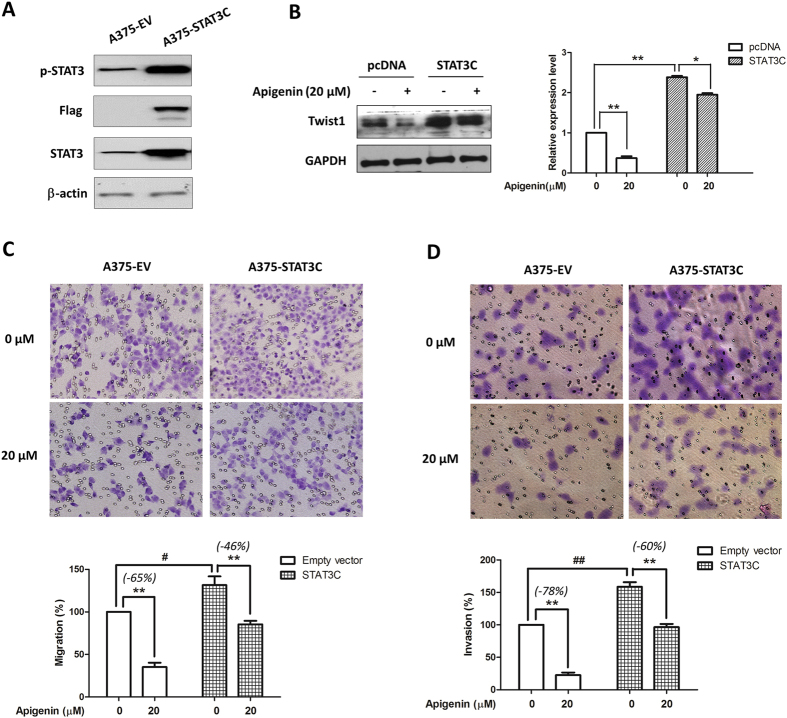
Overexpression of STAT3C reversed apigenin-mediated inhibition on cell migration and invasion, and reduced the apigenin-mediated Twist 1 inhibition. (**A**) A375 cells were transiently transfected with an empty vector or a STA3TC-expressing construct for 24 h, and then lysed for the Western blot analysis by using antibodies specific to p-STAT3, Flag, STAT3 and β-actin. (**B**) After transfection, cells were treated with apigenin for 24 h, and the effects of apigenin on Twist1 were determined by immunoblotting. The relative expression levels were analyzed by Image J software and shown as mean ± S.D., *P < 0.05, **P < 0.01. (**C**) Cell migratory and (**D**) invasive capacities were measured by the Transwell chamber assay. Representative photographs of migrated or invasive cells (upper) and quantification of these cells (bottom) were shown. Data were mean ± SD from three independent experiments, ^#^*P* < 0.05 ^##^*P* < 0.01 and **P < 0.01.

To further confirm if STAT3 inhibition was crucial in apigenin-mediated anti-migratory and anti-invasive activities, A375 cells were transiently transfected with a STAT3C-expressing construct before apigenin treatments. Western blotting data showed that transient transfect of the STAT3C-expressing construct in A375 cells resulted in a remarkable increase in STAT3 and p-STAT3 expressions (Fig. 7A). As expected, overexpression of STAT3 in A375 cells apparently increased the cell migratory (Fig. 7C) and invasive (Fig. 7D) abilities. And in STAT3C-expressing A375 cells, the inhibitory effects of apigenin on cell migration and invasion were inhibited from 65% to 46% and from 78% to 60%, respectively.

We also found that Twist1, a downstream target of STAT3^32^, was increased in STAT3-overexpressed A375 cells, and the apigenin-mediated Twist1 inhibition was also partially reversed (Fig. 7B). Moreover, the overexpression of STAT3 and hence the increased expression of Twist1 reduced the apigenin-mediated EMT suppression (see Supplementary Fig. 2).

Taken together, we suggest that inhibition of STAT3 activation is critical in apigenin-afforded anti-metastatic activity in melanoma.

## Discussions

Our research group is interested in the possible beneficial biological effects of natural compounds on malignant diseases, especially on melanoma^36^,^37^,^38^. In this study, we demonstrated the anti-metastatic effect of apigenin, a natural dietary compound that commonly presented in human diet, in melanoma cells and in animals. Our data showed that apigenin inhibited murine melanoma B16F10 cell lung metastasis in an animal model. Apigenin also exerted anti-migratory and anti-invasive activities in cultured B16F10 cells and human melanoma A375 cells. These results are in agreement with previous studies^19^,^20^.

Apigenin is generally non-toxic unless it is intraperitoneally injected with a high dose^39^. Indeed, apigenin in particular gains interest compared with other structurally related flavonoids because of its low intrinsic toxicity and its striking effect on cancer cells *versus* normal cells^40^. The average intake of flavonoids including apigenin, quercetin, kaempferol, myricetin and luteolin ranges from 6 mg/day in Finland to 64 mg/day in Japan^17^. Apigenin is emerging as one of the key nutraceuticals^21^. Our *in vivo* study also suggests a low toxicity of apigenin because we did not observe any apparent significant difference in body weights between apigenin-treated mice and control mice (see Supplementary Fig. 3).

The major problem for using apigenin as a chemopreventer is its poor oral bioavailability^41^. In 2005, Gradolatto *et al.* showed that oral intake a single dose of radio-labeled apigenin in rats resulted in 51% recovery of radioactivity in urine, 12% in feces, 1.2% in blood, 0.4% in the kidneys, 9.4% in the intestine, 1.2% in liver and 24.8% in the rest of the body within 10 days. The radioactivity did not appear in blood until 24 h after oral apigenin intake. The kinetics of apigenin in blood exhibited a relatively high elimination half-time of 91.8 h compared to other dietary flavonoids. These data suggest that although the bioavailability of apigenin is limited, the slow absorption and slow elimination properties may lead to possible accumulation of apigenin in the tissues to effectively impart its chemopreventive effects^42^. Indeed, in our study, oral administration with apigenin for 24 consecutive days significantly inhibited melanoma lung metastasis in mice. Daily intragastric injection of apigenin has also been reported to inhibit the progression and metastasis of ovarian cancer and prostate cancer in animal models^26^,^43^. Thus, daily oral intake of apigenin is able to prevent tumor progression and metastasis. Meanwhile, to improve the dissolution rate and increase the bioavailability of apigenin, several formulation techniques for apigenin have been investigated, including nanocrystals^44^, liposome^45^, and carbon nanopowder solid dispersion^46^. The solubility and bioavailability of apigenin have been significantly enhanced by these strategies. Taken together, apigenin is a promising anti-cancer agent.

The mechanistic study clearly showed that apigenin inhibited STAT3 activation, and moreover, cell migratory and invasive inhibitory effects of apigenin could be rescued by STAT3C (a constitutive active form of STAT3) over-expression. These data strongly suggest that inactivation of STAT3 signaling by apigenin may serve as an effective approach for melanoma treatment. After phosphorylation at Tyr-705, STAT3 dimerizes and translocates into the nucleus which in turn binds to the specific DNA response element in the promoter regions of target genes, leading to transcriptional activation. Therefore, inhibition of STAT3 phosphorylation may result in decreased STAT3 nuclear localization and transcriptional activity. In good agreement with this, our results indeed showed that STAT3 nuclear localization and transcriptional activity were substantially reduced by apigenin treatment. Constitutive STAT3 activation has been linked to the development of various cancer types. The suppression of constitutive STAT3 activation by apigenin in melanoma cells raises a possibility that apigenin may also inhibit the constitutively activated STAT3 in other cancer types, thereby providing a rationale for its use to treat other cancers. It is interesting to note that apigenin has been recently found to suppress STAT3 signaling in several types of cancer cells^22^,^23^.

Aberrant STAT3 activation is predominantly due to persistent tyrosine receptor kinase (Tyr) phosphorylation signals emanating from dysregulated upstream Tyr kinases, including Src and JAK2^30^. Our results showed that expressions of both phospho-JAK2 and phospho-Src were decreased in A375 and G361 cells. On the other hand, numerous protein tyrosine phosphatases (PTPs) such as SHP-1, SHP-2, TC-PTP and PTP-1D have been implicated in STAT3 signaling, and inhibition of STAT3 activation has also been linked to the induction of PTPs by some anticancer agents^47^. Whether PTPs are involved in apigenin-mediated STAT3 inactivation in cancer cells including melanoma cells remains unknown and deserves further investigation.

We showed that apigenin impeded A375 melanoma cell migration and invasion. MMP-2 and MMP-9 are collagenases which favor tumor growth and invasion by digesting the extracellular matrix surrounding the tumor tissue. Strong MMP-2 expression is prevalent in primary and metastatic melanomas as compared to normal and dysplastic nevi, and is associated with worse survival of melanoma patients^48^. High serum level of MMP-9 is associated with rapid progression in patients with metastatic melanoma^49^. Moreover, it has been reported that overexpression of STAT3 enhances the invasiveness in less-invasive melanoma cells through increasing MMP-2 expression and activity; while inactivation of STAT3 remarkably impairs the invasive ability of invasive melanoma cells through decreasing MMP-2 expression and activity^13^. In this regard, it is plausible to postulate that apigenin inhibited melanoma invasiveness by decreasing MMP-2 activity and expression through suppressing the constitutively active STAT3. Apigenin also decreased the activity of MMP-9. However, the basal expression level of MMP-9 was very low, which was in line with the others’ studies^8^.

The development of metastasis has been shown to depend on the development of an adequate blood supply through angiogenesis^50^. VEGF is the most potent pro-angiogenic stimulus that plays critical role in tumor angiogenesis and metastasis. In melanoma patient, high serum VEGF values are associated with shorter disease-free survival as compared with lower values^51^. STAT3 has been reported to directly participate in regulating transcription of the *VEGF* gene^9^. Inhibition of STAT3 signaling suppresses Src- and IL-6-medaited VEGF up-regulation as well as tumor angiogenesis^9^,^52^. In our study, the expression of VEGF in melanoma cells was decreased by apigenin treatment, suggesting that the anti-metastatic effect of apigenin on melanoma is associated with VEGF inhibition. Besides promotes tumor metastasis, STAT3 also participates in the immune evasion process through up-regulation of immunosuppressive factors and down-regulation of pro-inflammatory cytokines and pro-inflammatory chemokines^53^. VEGF is one of the immunosuppression factors^54^. Whether apigenin enhances the anti-melanoma immune responses through STAT3/VEGF inhibition needs to be further studied.

We also demonstrated that apigenin treatment reduced the expression of Twist1, another target gene of STAT3, which is generally overexpressed in melanoma^55^. However, the role of Twist1 in melanoma is less studied. It is reported that Twist1 promoted melanoma metastasis and growth, which was accompanied by the up-regulation of several vascular growth factors and receptors, including VEGF and MMP-1^55^,^56^. MMP-2 and MMP-9 can also be regulated by Twist1 and involved in Twist1-induced promotion of metastasis^57^,^58^. Both VEGF and MMPs are the molecular targets of STAT3. In this study, we suggest that Twist1 and STAT3 activation contribute to melanoma migratory and invasive abilities because overexpression of Twist1 or STAT3 enhanced cell migratory and invasive abilities, and also reversed apigenin-mediated melanoma migration and invasion inhibition.

EMT has been widely recognized as an important mechanism for cancer progression. Our data suggest that apigenin treatment reduces the expression of Twist1 and regulates EMT factors. Recently, a study suggests that invasion promoted by Twist1 is not due to a paradigm EMT protein changes in melanoma^59^. We will further investigate if the changed expressions of EMT markers, due to apigenin treatment or knockdown of Twist1 by siRNA, contribute to the inhibition of invasion in the melanoma cell model.

In conclusion, our study shows that inhibition of the STAT3 signaling pathway contributes to the anti-metastatic effect of apigenin in melanoma. Together with the reported anti-proliferative activity and low toxicity property of this compound, we suggest that apigenin has a potential role in melanoma treatment/prevention.

## Materials and Methods

### Reagents

Antibodies against phospho-STAT3 (Tyr705), STAT3, phospho-JAK2 (Y1007/1008), JAK2, phospho-Src (Tyr 416), Src, Keratin and Fibronectin were obtained from Cell Signaling Biotechnology (Beverly, MA, USA). Antibodies against Twist1 and β-actin, and Twist1 small interfering RNA (Twist1-siRNA) were purchased from Santa Cruz Company. Goat anti-rabbit IgG, goat anti-mouse IgG and protein marker were supplied by Bio-Rad (Hercules, CA, USA). Antibody against N-cadherin was purchased from BD Biosciences (San Jose, CA, USA). Other chemicals were obtained from Sigma-Aldrich (St. Louis, MO, USA). Apigenin was obtained from Shanghai R&D Center for Standardization of Chinese Medicines (Shanghai, China, purity >99% as determined by HPLC). The stock solution of 100 mM apigenin was prepared in dimethyl sulfoxide (DMSO).

### Cell culture

Human melanoma A375 and G361 cell lines, and murine melanoma B16F10 cells were purchased from the American Type Culture Collection (ATCC, USA) and maintained in high glucose Dulbecco’s modified Eagle’s medium (DMEM, GIBCO, USA) supplemented with 10% fetal bovine serum (FBS, GIBCO, USA) and 1% penicillin/streptomycin (P/S, GIBCO, USA) in a humidified 5% CO_2_ atmosphere at 37 °C.

### Wound healing assay

Wounds were created by scratching the confluent cell monolayer using a plastic pipette tip, and any loose cellular debris or detached cells were removed by PBS wash. The cells were then refed with full DMEM medium containing DMSO or apigenin with mitomycin C (Sigma-Aldrich) to block mitosis. The gaps of the wounds were observed with phase contrast microscopy and digitally photographed under 100× magnifications. Each experiment was performed in triplicate.

### Transwell migration assay

Transwell migration assay was performed using 6.5 mm diameter transwell cell culture chamber units (Costar, Cambridge, MA) with 8-μm pore size polycarbonate membranes. Briefly, cells (5 × 10^4^) suspended in DMEM-0.1% BSA with apigenin or DMSO were plated in the upper chamber wells, while serum-starved NIH-3T3 culture medium was added to the lower chamber wells as chemoattractants. After incubation for 16 h at 37 °C, cells in upper wells were completely removed from the upper surface of the filters with cotton swabs. Cells that had migrated to the lower surface of the filters were fixed with methanol and stained with crystal violet. The migratory phenotypes were determined by counting the cells that migrated to the lower side of the filters in different fields under a microscope at 200×. Assays were repeated in triplicate.

### Transwell invasion assay

Cell invasion was determined by using BD BioCoat™ Matrigel™ invasion chamber (BD Biosciences) according to the manufacturer’s instruction. In brief, 0.75 ml of serum-starved NIH-3T3 culture medium was added to the lower chamber wells as chemoattractants, and aliquots of 0.5 ml of cell suspensions consisting of 5 × 10^5^ cells/ml in DMEM-0.1% BSA containing apigenin or DMSO were seeded on upper wells and allowed to invade for 16 h. The non-invading cells were removed by scrubbing with cotton swab and the cells on the lower surface of the membrane were stained with crystal violet. The invasive capacity was quantified by counting the cells that migrated to the lower side of the filters in different fields under a microscope at 200×. Assays were repeated in triplicate and data were expressed as the percentage of invasion in control.

### Western blotting

Western blot analysis was performed as described previously^60^. Briefly, cells were harvested and lysed with the RIPA buffer (50 mM Tris-HCl, 1% NP-40, 0.35% sodium-deoxycholate, 150 mM NaCl, 1 mM EDTA, pH 7.4, 1 mM phenylmethylsulfonyl fluoride, 1 mM NaF, 1 mM Na_3_VO_4_ and 10 μg/ml each of aprotinin, leupetin and pepstatin A). Equal amount of protein samples were separated by sodium dodecyl sulfate-poly-acrylamide gel electrophoresis (SDS-PAGE) and then electro-transferred onto nitrocellulose membranes (Amersham Biosciences, USA). After blocked with 5% milk in the TBST buffer for 1 h, membranes were subsequently probed with appropriate primary antibodies overnight at 4 °C. The membranes were then incubated with HRP-conjugated secondary antibodies. Immunoreactive bands were visualized using the ECL detection kit (Invitrogen, USA).

### Semi-quantitative and real-time quantitative polymerase chain reaction analyses

Total RNA was extracted with Trizol reagent (Invitrogen, USA), and reverse-transcribed with oligo-dT using the M-MLV reverse transcriptase (Promega, USA) according to the manufacturer’s protocol. For semi-quantitative polymerase chain reaction (PCR), the resultant cDNA was subjected to 25–30 cycles of PCR amplification (denaturing at 95 °C for 30 s, annealing at 55–60 °C for 30 s, extension at 72 °C for 60 s). The PCR products were separated by electrophoresis on a 2% agarose gel and visualized with ethidium bromide staining. Quantitative real-time PCR was performed using SYBR green reaction mixture in the ABI 7500 fast real-time PCR system (Applied Biosystems, USA). The PCR conditions were one cycle at 55 °C for 2 min and at 95 °C for 10 min, followed by 40 cycles of amplification at 95 °C for 15 s and at 60 °C for 1 min. The fluorescent signals were detected using the ABI Prism 7500HT sequence detection system (Applied Biosystems, USA). The gene expression data were normalized to the endogenous control β-actin. The relative expression levels of genes were measured according to the formula 2^−ΔΔCt^, where ΔCt is the difference in threshold cycle values between the targets and β-actin, and ΔΔCt is the difference between the treatment and vehicle control groups. All samples were analyzed in triplicate.

### Gelatin zymography

Cells were treated with apigenin or vehicle for 24 h and then cultured in serum-free medium for 24 h. Twelve hours later, the serum-free supernatants were harvested and concentrated using Centricon YM-10 concentrator (Millipore, USA). After the protein concentrations were determined, the samples were separated by SDS-PAGE in 10% polyacrylamide gel containing 0.1% gelatin under non-reducing conditions. Following electrophoresis, the gels were washed in renaturing buffer (50 mM Tris-HCl, pH 7.5, and 2.5% Triton X-100), and incubated overnight in developing buffer (50 mM Tris-HCl, pH 7.5, 5 mM CaCl_2_, 200 mM NaCl, 0.2% Brij-35) to allow the gelatinases to digest the gelatin structure. The gels were stained with 0.5% Coomassie Brillant Blue R-250 in 30% methanol and 10% acetic acid. The gelatinase activity was visible as clear bands on blue background.

### Immunofluorescence staining for STAT3 localization

Cells were seeded on glass coverslips in 6-well plate overnight, and treated with apigenin or vehicle for 24 h. Cells were then fixed with 4% paraformaldehyde and permeablized with 0.5% Triton X-100 in PBS. After blocked with 5% bovine serum albumin (BSA) in PBS, the cells were incubated with an anti-STAT3 antibody diluted in PBS/2.5% BSA (1:500) overnight. Subsequently, the cells were washed with PBS followed by incubation with Cy2-conjugated anti-rabbit secondary antibody (Invitrogen, USA) diluted in 2.5% BSA/PBS (1:400) for 1 h. Cells were washed in PBS and counterstained with DAPI (Sigma-Aldrich, USA). Images of the cell signal were captured by a fluorescence microscope.

### Plasmids

We generated Twist1 promoter reporter construct Twist1-Luc by cloning human Twist1 promoter −451 to +1 fragment into pGL3-basic luciferase vector at KpnI/XhoI sites. The full length Twist1 coding domain sequence was PCR amplified from pWZL Blast Twist1 ER construct (Addgene, USA) and inserted into pcDNA3.1 vector at BamHI/ECORI sites, yielding pcDNA3.1-Twist1. The STAT3 reporter construct 4xM67 pTATA TK-Luc and the constitutive activated STAT3 expression construct STAT3-C Flag pRc/CMV were obtained from Addgene.

### Transfection

Transfection of plasmids and siRNAs into melanoma cells was conducted by using Lipofectamine 2000 (Invitrogen, USA). Cells were transfected with indicated siRNA or plasmids for 24 h or 48 h before functional assays were carried out.

### Luciferase reporter assay

Cells were seeded in 24-well plates and co-transfected with the reporter construct (Twist1-Luc or 4xM67 pTATA TK-Luc) plus *Renilla* luciferase expression vector PRL-CMV (Promega, USA) by using lipofectamine 2000 reagent (Invitrogen, USA). At 12 h post-transfection, cells were treated with apigenin or vehicle (DMSO) for another 12 h. Then cells were harvested for luciferase reporter assay using the dual-luciferase reporter assay system (Promega, USA). Firefly luciferase values were normalized to *Renilla* luciferase values. All assays were performed in triplicate.

### Experimental lung metastasis

C57BL/6 mice were obtained from The Laboratory Animal Services Centre, The Chinese University of Hong Kong. Lung metastasis of B16F10 mouse melanoma was done as described elsewhere^61^. Briefly, B16F10 melanoma cells were washed in PBS containing 5 mM EDTA, the cell number and viability were examined using trypan blue. Sample containing only single-cell suspension of >90% viability were suspended at 3 × 10^5^ cells in 50 μl PBS and were injected into the tail vein of the C57BL/6 mice. These mice were then randomly divided into 2 groups and received intragastric administration of either 0.5% CMC-Na solution as the vehicle control or apigenin (150 mg/kg/day)^26^ for consecutive 24 days. After the treatment, mice were scarified and lungs were dissected. Metastasized colonies were counted using a dissecting microscope.

### Statistical analysis

Data were summarized as mean ± SD. The significant difference between two groups was analyzed using the Student’s *t*-test.

## Additional Information

**How to cite this article**: Cao, H.-H. *et al.* Inhibition of the STAT3 signaling pathway contributes to apigenin-mediated anti-metastatic effect in melanoma. *Sci. Rep.* **6**, 21731; doi: 10.1038/srep21731 (2016).

## Supplementary Material

Supplementary Information
