# Supplementary material for: Inhibition of the STAT3 signaling pathway contributes to apigenin-mediated anti-metastatic effect in melanoma
Source: Sci Rep. 2016 Feb 25;6:21731. doi: 10.1038/srep21731 (PMC4766576; doi:10.1038/srep21731)
Supplement: Supplementary Information [file srep21731-s1.pdf]

Inhibition of the STAT3 signaling pathway contributes to apigenin-mediated anti-metastatic effect in melanoma

Hui-Hui Cao[1,+], Jian-Hong Chu[1,+], Hiu-Yee Kwan[1,+], Tao Su[1], Hua Yu[1], Chi-Yan Cheng[1], Xiu-Qiong Fu[1], Hui Guo[1], Ting Li[1], Anfernee Kai-Wing Tse[1], Gui-Xin Chou[2], Huan-Biao Mo[3], Zhi-Ling Yu[1,]*

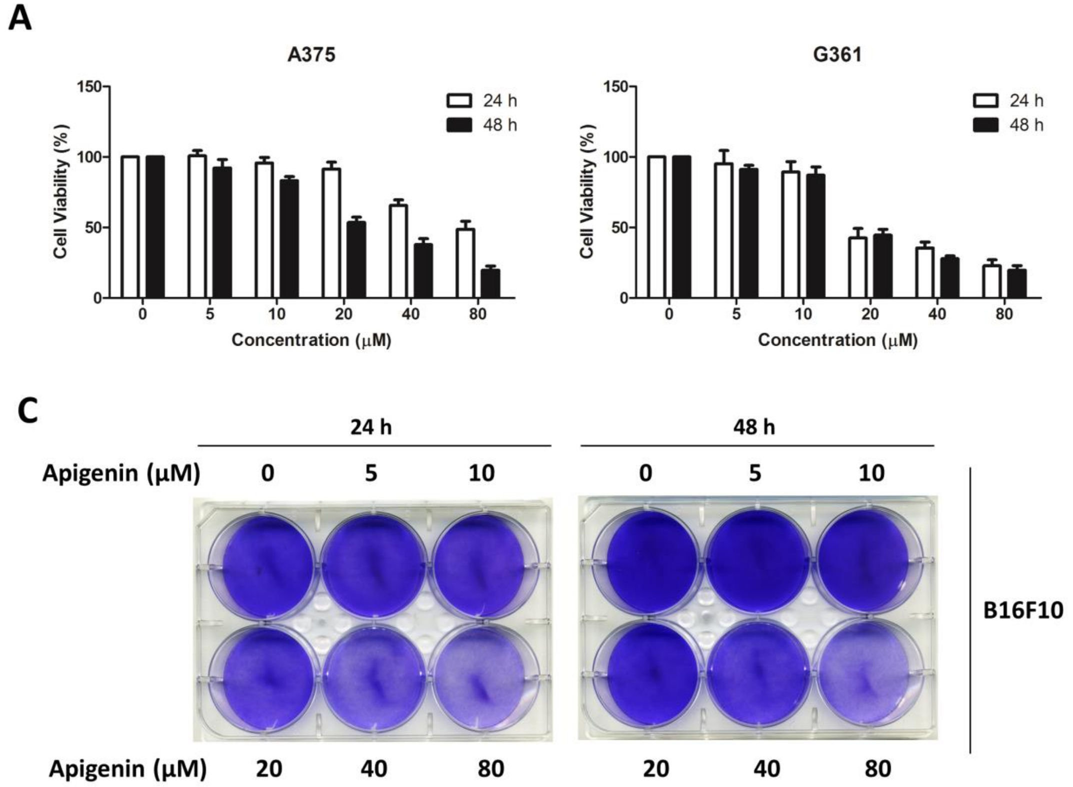

Supplementary figure 1. Effect of apigenin on melanoma cells proliferation. (A) A375 and G361 cells were treated with various concentrations of apigenin for 24 h or 48 h. Cell viability was measured by the MTT assay and viability curves were shown. (B) B16F10 cells were treated with various concentrations of apigenin for 24 h or 48 h, cell viability was measured by the crystal violet staining assay and the representative photographs from three independent experiments were shown.

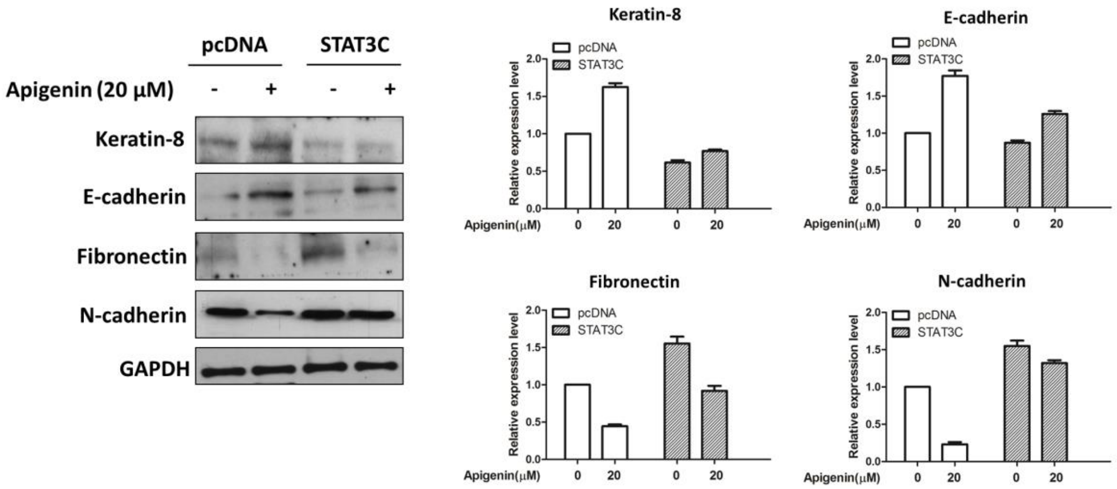

Supplementary figure 2. Overexpression of STAT3 reduced the apigenin-mediated EMT suppression in A375 cells. After transiently transfected with either an empty vector or a STA3TC-expressing construct for 24 h, A375 cells were treated with apigenin for 24 h, and the effects of apigenin on Keratin-8, E-cadherin, Fibronectin and N-cadherin were determined by immunoblotting. The representative results (left) relative expression levels (right) were shown.

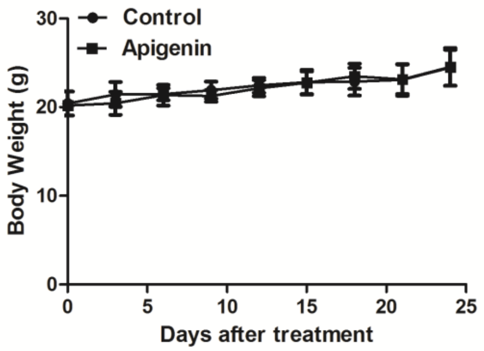

Supplementary figure 3. Changes in body weights of mice after apigenin treatment. Body weights were measured every three days and presented as the mean ± SD, *n*=6.
